# Supplementary material for: Nb2O5 Coating to Improve the Cyclic Stability and Voltage Decay of Li-Rich Cathode Material for Lithium-Ion Battery
Source: Molecules. 2023 May 5;28(9):3890. doi: 10.3390/molecules28093890 (PMC10179934; doi:10.3390/molecules28093890)
Supplement: Supplementary file 1 [file molecules-28-03890-s001.zip › molecules-2290835-supplementary-update.pdf]

Supplementary Information

Nb_2O_5 Coating to Improve the Cyclic Stability and Voltage Decay of Li-Rich Cathode Material for Lithium-Ion Battery

Yanlin Liu [1,2], Ruifeng Yang [1], Xinxi Li [1,*], Wensheng Yang [1], Yuanwei Lin [2], Guoqing Zhang [1] and Lijuan Wang [3,*]

1 School of Material and Energy, Guangdong University of Technology, Guangzhou 510006, China

2 School of Automobile and Transportation Engineering, Guangdong Polytechnic Normal University, Guangzhou 510640, China

3 College of Petroleum and Chemical Technology, Liaoning Petrochemical University, Fushun, 113001, China

* Correspondence: pkdlxx@163.com (X.L.); lijuanw123@163.com (L.W.)

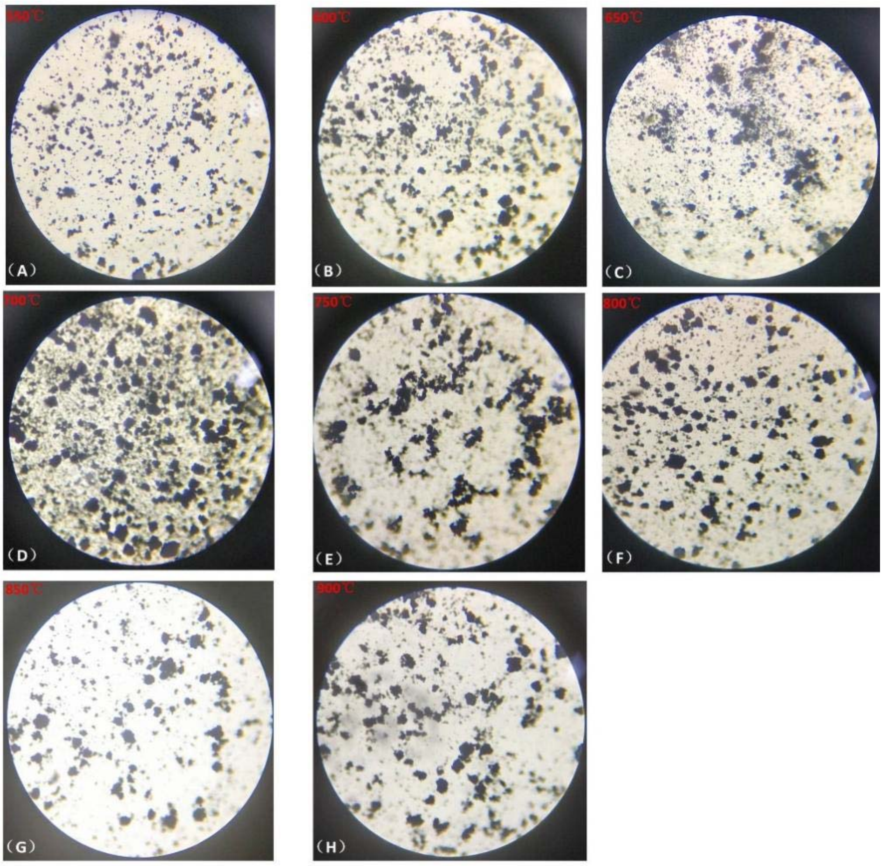

Figure S1. The microscope images of samples obtained at 550 ℃, 600 ℃, 650 ℃, 700 ℃, 750 ℃, 800 ℃, 850 ℃, and 900 ℃, respectively.

Figure S1 shows the microscope images of samples obtained at 550 ℃, 600 ℃, 650 ℃, 700 ℃, 750 ℃, 800 ℃, 850 ℃, and 900 ℃, respectively when the coating ratio is 0.010. It can be seen from that the material shows agglomeration, and the particle size increase as the sintering temperature increasing. This may be due to the gradual growth of material grains when the sintering temperature increase.

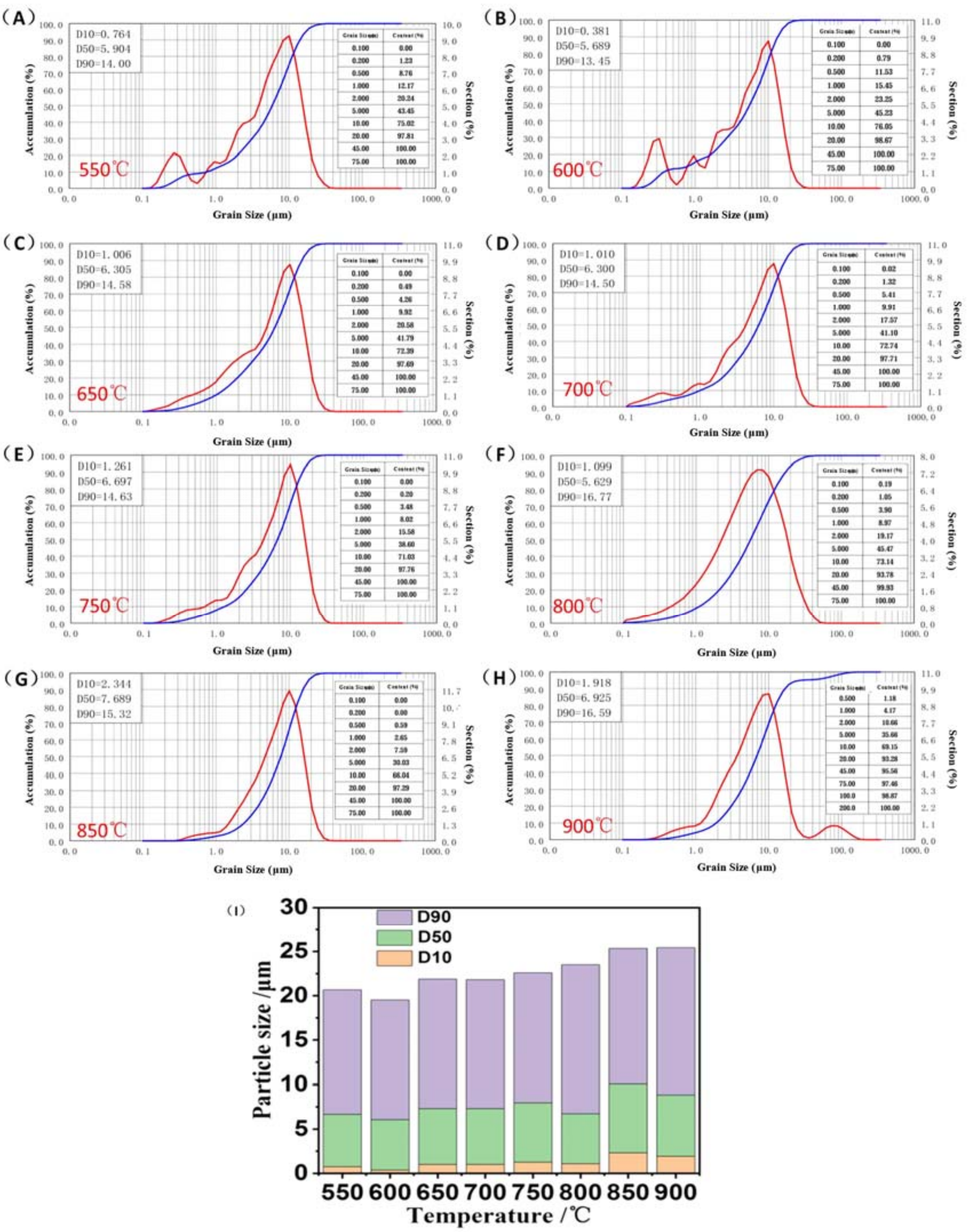

Figure S2. The particle size of samples obtained at 550 °C, 600 °C, 650 °C, 700 °C, 750 °C, 800 °C, 850 °C, and 900 °C, respectively.

As shown in Figure 4.5. It can be found that the particle size D10, D50 and D90 gradual increase with the increasement of sintering temperature, and reach the highest at 850 °C. It should be notice that there is a reverse when it rises to 800 °C. Further analysis can be found that many small particles with extensive diameter are exist at the range of 550 °C−750 °C, while these small peaks were vanished when the temperature

raise up to 800 °C. It suggests that the 800 °C is the crystallization temperature and the small particle size gradually disappeared as the crystal grains gradually grow. However, the crystal lattice of the RLM material maybe to be changed when the temperature further be raised up to 900 °C, thus showing a third−order particle distribution as shown in Figure S2 (H).

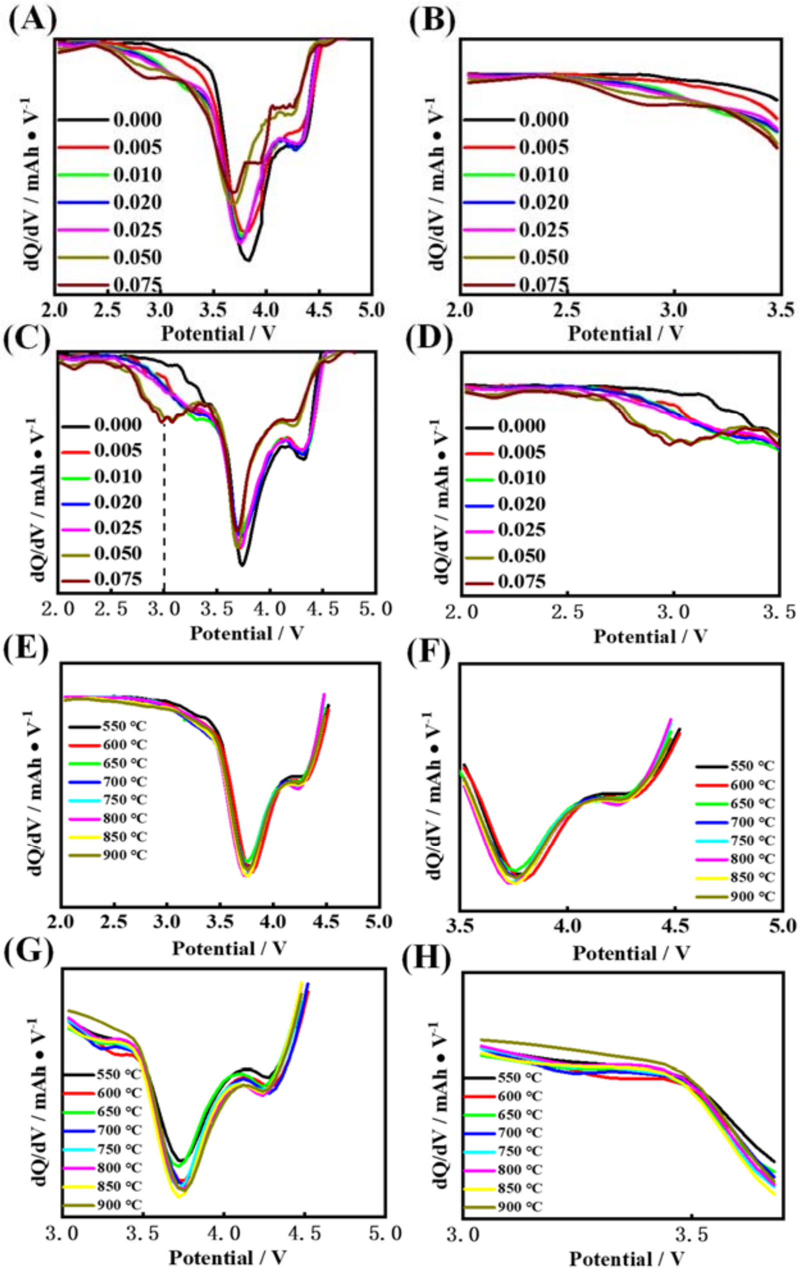

Figure S3. dQ/dV curves of the initial cycle (A−B), and dQ/dV curves of the 8th cycle (C−D) for different coating ratios of RLM@Nb_2O_5, dQ/dV curves of the 1th cycle (E)−(F) and 8th cycle (G)−(H) of the samples obtained at550 °C, 600 °C, 650 °C, 700 °C, 750 °C, 800 °C, 850 °C, and 900 °C.

As show in Figure 1, 2, 3 and, S3, the peaks located between 2.7−3.0 V and correspond to the reduction of Mn^{4+} in the spinel phase in the lamellar structure. Therefore, the discharge current appears in the range of 2.75−3.0 V, which was regarded as belongs to the spinel structure formed by the transfer of Mn, and becomes larger as the increasement of Nb_2O_5 amount. Additionally, the current peak around 3.7 V shows blue shift when the Nb_2O_5 increases, this may due to the introduced resistant by too thick Nb_2O_5 layer. Notably, the larger response current below 3.6 V of the 0.050 and 0.075 coating ratios than others were shown, this can be ascribed to the excessive formed spinel phase caused by the more Nb_2O_5. Moreover, the huge loss of the layered structural component was implicated in the excessive spinel phase forming, result in the smaller current in the range of 3.8−4.6 V. The obviously positive shift of the charging peaks means the larger polarization that caused by the excessive Nb_2O_5, when the coating ratio is above 0.050.
